# Supplementary material for: Causal Relationship between Aging and Anorexia Nervosa: A White-Matter-Microstructure-Mediated Mendelian Randomization Analysis
Source: Biomedicines. 2024 Aug 16;12(8):1874. doi: 10.3390/biomedicines12081874 (PMC11351342; doi:10.3390/biomedicines12081874)
Supplement: Supplementary file 1 [file biomedicines-12-01874-s001.zip › Supplementary Figure/Figure S8/Leaveoneout plot of MR analysis for TL to ubm-b-2114.pdf]

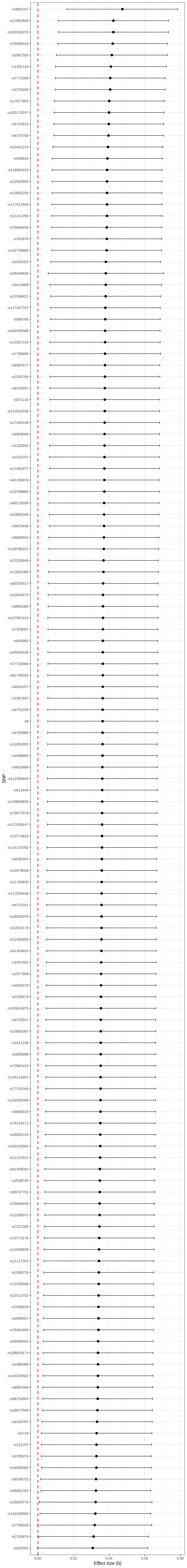

rs3891167
rs17803849
rs202034370
rs76065543
rs2967355
rs1291143
rs7772289
rs2276182
rs17677991
rs185174247
rs5742915
rs6776756
rs12412214
rs939916
rs116863223
rs12925933
rs10905255
rs117512405
rs11412296
rs75664430
rs762679
rs142730696
rs1003322
rs35446936
rs9419958
rs11584821
rs117407747
rs869785
rs182059586
rs11557154
rs7790856
rs6587577
rs2282764
rs8102497
rs871134
rs113525195
rs17445108
rs9940099
rs1332941
rs1023767
rs11991877
rs41269079
rs10768683
rs80116508
rs10805346
rs9878436
rs6669563
rs139795227
rs13230646
rs13062095
rs80324517
rs10840270
rs9955360
rs137901416
rs7209057
rs450962
rs45604339
rs77732866
rs61748181
rs6054257
rs1957937
rs6751209
All
rs6790988
rs12451892
rs4498805
rs4616688
rs112394943
rs611646
rs139669835
rs79977579
rs117630647
rs10774624
rs141214782
rs6590343
rs16978028
rs11769630
rs117034449
rs4731541
rs28363070
rs12932179
rs12369950
rs41304832
rs3767952
rs2977608
rs4530278
rs2293579
rs376641875
rs4743037
rs10845387
rs1611236
rs3093888
rs73581419
rs145114957
rs77231040
rs142426306
rs9600019
rs76219171
rs28502153
rs150150565
rs11212631
rs61405042
rs2538745
rs55747751
rs76666449
rs11085072
rs7221585
rs10773176
rs11699829
rs11117354
rs2056726
rs73730598
rs10112752
rs7099229
rs4695407
rs78491606
rs59409453
rs188918174
rs1985369
rs144204502
rs6881568
rs66731853
rs28577594
rs8105767
rs4724
rs131797
rs3785074
rs34550383
rs6536702
rs56061761
rs35640778
rs143190905
rs7705526
rs2763979
rs932002
SNP
0.00
0.02
0.04
0.06
0.08
Effect size (b)
